# Digital Twin for a Frequency Mixer Used as a Phase Sensor

**DOI:** 10.3390/s24237574

**Published:** 2024-11-27

**Authors:** Carlos Pires, Manuel Abreu, Isabel Godinho, Rui Agostinho, João A. Sousa

**Affiliations:** 1 Instituto Português da Qualidade, Rua António Gião, 2, 2829-513 Caparica, Portugal; carlosp@ipq.pt (C.P.); igodinho@ipq.pt (I.G.); jasousa@ipq.pt (J.A.S.); 2 Physics Department, Faculdade de Ciências da Universidade de Lisboa, 1749-016 Lisboa, Portugal; rjagostinho@ciencias.ulisboa.pt

**Keywords:** digital twin, phase measurements, metrology, traceability, frequency mixer

## Abstract

The Portuguese Institute for Quality is responsible for the realization and dissemination of the frequency standard in Portugal. There are several techniques for frequency transfer, but we use a frequency mixer to detect phase variations between two light signals with different wavelengths, traveling along an optical fibre. In this paper, we present the development of a digital twin (DT) that replicates the use of a frequency mixer to improve the frequency transfer problem. A setup was built to train and validate the technique: a frequency mixer was used to determine the phase difference between the two signals, which are caused by temperature gradients in the fibre, together with real-time temperature data from sensors placed along the fibre and on the mixer itself. The DT was trained with two machine learning algorithms, in particular, ARIMA and LSTM networks. To estimate the accuracy of the frequency mixer working as a phasemeter, several sources of uncertainty were considered and included in the DT model, with the goal of obtaining a phase value measurement and its uncertainty in real time. The JCGM 100:2008 and JCGM 101:2008 approaches were used for the estimation of the uncertainty budget. With this work, we merge DT technology with a frequency mixer used for phase detection to provide its value and uncertainty in real time.

## 1. Introduction

Metrology day is celebrated annually on May 20th, and in 2022 the theme was “Metrology in the Digital Era”. This theme highlights the importance of digital technology, such as Digital-SI, Big Data, Artificial Intelligence, and machine learning (ML), which are revolutionizing metrology as some of the most exciting societal trends of today. In 2020, the European Commission published the strategy ‘Shaping Europe’s digital future’, alongside its European Industry Strategy, Data Strategy, and the White Paper on Artificial Intelligence. As stated in a joint message by the directors of the Bureau International des Poids et Measures (BIPM) and International Bureau of Legal Metrology (BIML): “The digital transformation of metrology can bring many benefits to our community. It can expedite time to market for measurement products and services and reduce delays associated with approval processes. In turn, this contributes to innovation, product agility and sustainability” [1].

In this context, all National Metrology Institutes (NMIs) were encouraged to apply these “new” digital tools to metrology. As the Portuguese National Metrology Institute, the Portuguese Institute for Quality, IPQ, is responsible for the realization and dissemination of the national reference time scale, UTC(IPQ). Thus, digital transformation has begun in the Laboratory of Time and Frequency (LTF), where we are implementing a digital twin (DT) to measure phases using a frequency mixer. Transferring the standard frequency allows users to syntonize their local oscillators to the reference time scale. Of the existing techniques for frequency transfer, one uses a frequency mixer for relative phase-difference detections [2], which allows corrections in the process of frequency transfer with the objective of increasing the frequency stability at the end-user level. Applying these new digital tools to frequency transfer is one way to give traceability to the synchronization of oscillators.

In many research projects, simulations serve as a tool to replicate various system processes, enabling analysis and optimization at lower costs. However, traditional simulations often rely on theoretical models and lack real data. The concept of the digital twin, developed in recent years, addresses these limitations. While it utilizes the same mathematical models as simulations, a digital twin integrates real-world data, employs machine learning methods for prediction, and incorporates a user-friendly interface. This creates a significantly richer and more dynamic environment for studying and optimizing systems.

In communication systems, phase detection is essential for synchronizing signals. A frequency mixer is a key component in phase detection, especially in systems like Phase-Locked Loops (PLLs), radio communications, and signal processing. Although some nonideal characteristics exist, theoretically, any frequency mixer could be used as a phase detector. In this mode, a mixer compares the phases of two input signals by multiplying them and producing an output that reflects their phase difference.

The primary objective of implementing a DT for phase detection is to enhance the prediction performance of an optical channel transmission, including all terminal equipment, when sending a frequency standard signal over fibre optics. Furthermore, the DT of a frequency transmission setup enables the evaluation of all devices’ stability by detecting phase fluctuations induced by thermal perturbations over the entire medium, with a level of accuracy compatible with the standards required in some applications. By accurately measuring the signal’s phase, temperature-induced fluctuations can be compensated for, thereby improving stability and reliability in the frequency transfer.

In general, the frequency stability of an oscillator in free mode and in small integration times is better than the frequency stability of a secondary oscillator at the end of a transport medium, the reason being that the medium becomes the biggest source of uncertainty to the secondary oscillator, regardless of its inherent stability or accuracy.

**Figure 1:**
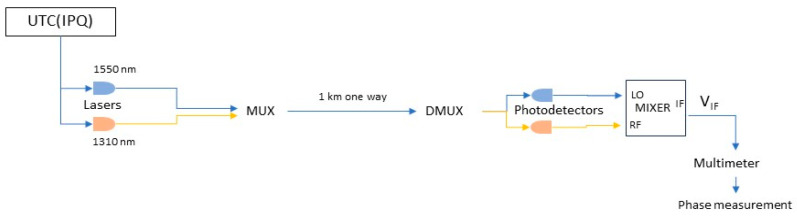
Representation of the physical system for phase measurement, which includes two lasers to transform the signal from UTC(IPQ) into light, two wavelength multiplexers (MUX and DMUX), two photodetectors to detect light, a frequency mixer, and a multimeter to measure voltage.

The experimental setup of this work is represented in Figure 1 and the physical part of it is a 1000 m optical fibre link between two laboratories. In one laboratory, a 10 MHz reference frequency is generated by three caesium (Cs) clocks with a stability value of the order of 5 × 10^−14^ for one day, traceable to the Coordinated Universal Time (UTC) designated as UTC(IPQ). The temperature and relative humidity conditions were measured and recorded at a rate of one measurement per minute.

The experimental setup includes the following equipment: two lasers with different wavelengths, namely 1310 nm and 1550 nm, two Bias-T instruments for amplitude modulation with the reference frequency, and a wavelength division multiplexer (WDM) to send both signals through the optical fibre. The 1000 m long optical fibre linking the two laboratories is standard in telecommunications (SMF-28, ETC, Portugal, 2021). The fibre runs on the rooftop of the building and is folded over, backward and forward, several times on the same path. In this way, more than 90% of the fibre is affected by the outside temperature.

In the second laboratory, we have a WDM for separating the light according to the wavelength, one photodetector for each of the wavelengths, a frequency mixer, and a multimeter to measure voltage at the output terminal of the mixer.

With the setup, we can correlate the mixer’s output voltage with temperature gradients in the optical fibre.

The development of the DT followed these key concepts:

- Final goal—predict a phase value with respective uncertainty.
- Collect data—gather data from all sensors along the system.
- Create a virtual model of the transit time of the light signals and the frequency mixer.
- Connect physical and digital model—obtain temperature values from the sensors every few minutes and predict phase values. This is a real-time interaction.
- Validate and test—several algorithms were used and validated.

The DT facilitates monitoring the parameters impacting frequency transfer delays and predicts corrective measures by adjusting these same parameters. All uncertainty contributions are assessed, with the DT calculating a phase value and its associated standard uncertainty as outputs.

## 2. Frequency Mixer for Phase Measurement: Principles

A frequency mixer is a nonlinear electrical circuit that generates new frequencies from two input signals. In its simplest form, it takes two applied signals and produces new signals equal to the sum and difference of the original frequencies. Additionally, other frequency components can be generated in a standard frequency mixer. The primary use of frequency mixers is for heterodyning signals, but they can also serve as phase detectors [3].

The basic concept upon which phase detection rests is that applying two identical frequencies with constant amplitude signals to a mixer generates a DC output proportional to the phase difference between the two signals.

There are different types of frequency mixers, but they all have the same basic connections. These frequency mixers have three ports, two inputs and one output, as follows:

- RF: This input is used for the signal whose frequency is to be changed. It is typically the incoming signal and normally of a relatively low level compared to the other input.
- LO: This is for the local oscillator signal. The input level here is generally much larger than the RF input.
- IF: This is the output port with the “mixed” signal.

For this work, we used a double-balanced mixer. Basic traditional double-balanced mixers typically use four Schottky diodes in a quad-ring configuration. The baluns or hybrids are placed at both the RF and LO ports, while the IF signal is tapped off from the RF balun [4]. When a double-balanced mixer is used as a phase detector and when we apply two in phase (frequency-matched) voltage signals to the LO and RF ports, the current will flow into the IF balun, creating a positive DC voltage at the IF port. When applying out-of-phase signals, the current is always pulled out of the IF balun, thus creating a negative DC voltage at the IF port. For quadrature signals, equal current flows both into and out of the IF balun; no DC current is created and no voltage is apparent.

For phase detection, we can assume that the output voltage (IF port) can be described as $V_{IF}=V\cos\left(∆\varphi +\pi \right)$, where *V* is the amplitude of the maximum voltage observed at ∆*φ* = 0, *π*. Assuming that the frequency mixer is in its linear response region, with some mathematical manipulation we can obtain the following Equation [3]:

(1) $$V_{out}=V\left(∆\varphi -\frac{\pi }{2}\right)$$

From Equation (1) we obtain

(2) $$∆\varphi =\frac{V_{out}}{V}+\frac{\pi }{2}$$

Which relates phase, in radians, with the output voltage of the mixer.

## 3. Transit Time

The technique used for time transfer characterization is based on the group delay difference (transit time) between two different wavelength signals propagating in a single-mode fibre. They have different propagation speeds in the fibre. An important parameter to consider is the fibre’s temperature *T*, as the variation in transfer time is a function of it.

In this work, a single-mode optical fibre was used.

The two main effects degrading the signal, both critical to its performance, are attenuation and dispersion.

The time of flight (*tof*) through an optical fibre of length *L* for a wavelength *λ* is given by

(3) $$\tau_{p}=\frac{L\;n(\lambda )}{c}=tof$$

where *c* is the speed of light in vacuum and *n* the optical fibre’s refractive index.

The system is composed of two lasers with different wavelengths: 1310 nm and 1550 nm.

The difference between the propagation times for two different wavelengths is given by

(4) $${{d\tau }_{p}=\tau }_{p1}-\tau_{p2}=\frac{L}{c}\left(n_{1}-n_{2}\right)$$

From a physical point of view, the group delay time, *τ_g_*, i.e., the wave group *tof* along the fibre, is proportional to the derivative of the refractive index *n*(*λ*). This is the propagation time of a signal in a fibre, or the group delay time for a single-mode fibre [2]:

(5) $$\tau_{g}=\frac{L}{c}\left(n-\lambda \frac{dn}{d\lambda }\right)$$

From Equation (5), one concludes that different wavelengths will propagate at different velocities in the same fibre. The difference in the propagation time of two signals with different λ’s produces a phase difference between them.

The effect of temperature on an optical fibre is determined by changes in the variation in both of its length and refractive index. By measuring the temperature of the fibre, we can correlate and determine these phase variations.

Assuming that polarization mode dispersion is small compared to chromatic dispersion for the wavelengths of interest, we can dismiss this effect on the transit time. Deriving Equation (5) with respect to temperature and combining it for different wavelengths, we obtain the phase-difference temperature variation for two signals traveling throughout the fibre [2]:

(6) $${\left.\frac{d\tau }{dT}\right|}_{\lambda_{1}{-\lambda }_{2}}=\frac{1}{c}\left(\frac{dL}{dT}\left(\left(n_{\lambda_{1}}-n_{\lambda_{2}}\right)+\lambda_{2}\frac{dn_{\lambda_{2}}}{d\lambda_{2}}-\lambda_{1}\frac{dn_{\lambda_{1}}}{d\lambda_{1}}\right)+L\frac{d}{dT}\left(\left(n_{\lambda_{1}}-n_{\lambda_{2}}\right)+\lambda_{2}\frac{dn_{\lambda_{2}}}{d\lambda_{2}}-\lambda_{1}\frac{dn_{\lambda_{1}}}{d\lambda_{1}}\right)\right)$$

Equation (6) is included in the DT to calculate the theoretical phase. This will be discussed later in this paper.

## 4. Digital Twin

We consider a digital twin as a computerized version of a physical (or real) system that can be programmed to behave like the real system, enabling one to run scenarios and obtain a response to these scenarios. A DT is a technology that is more than just the digital representation of the real object; it also enables bi-directional data exchange and real-time management.

To create a digital twin, we need physical data, simulated data, and the interaction data between the two to map them together to produce a digital replica of the system, i.e., a digital representation of a physical object, process, or system as a numerical model. Real-time data, algorithms (simulation), and machine learning are used to create this virtual representation. This means that changes in the physical twin will be reflected in the virtual twin. The data collected by the digital part are strictly dependent on the physical part, which makes the digital twin very accurate.

In most cases, digital twins are built from five fundamental capabilities: sensors, data, integration, modelling/analysis, and actuation.

For this experiment, we intend to use this concept in the following way:

1. Transform the equipment that generates and sends the 10 MHz signal to the optical fibre into a digital twin. Copy into digital format (numerical models) the characteristics of the lasers plus their uncertainties, the 10 MHz signal plus its uncertainty, the signal modelling and temperature sensors associated with the lasers, and any other equipment.
2. Add the optical fibre to the DT, e.g., the refractive index of the optical fibre and the two multiplexers at the beginning and end of the fibre, and also the temperature sensor data that measure the fibre.
3. Likewise, on the reception part, include detectors, temperature sensors, the frequency mixer, and the equipment that measures the phase between the two signals that pass through the optical fibre.
4. Data from the temperature sensor and multimeter are measured simultaneously, stored in files, and retrieved by the DT.
5. Train and validate an algorithm to predict a phase value.
6. Data from every sensor or piece of measurement equipment have an associated uncertainty, which allows the knowledge of a phase value with its associated uncertainty.
7. The exchange data between the virtual part of the DT and the physical part consist of the simultaneous measurement, temperature, and voltage values.

This allows us to obtain a detailed simulation of the entire system, complemented with real data, and predictions for phase values.

The use of ML in digital twins is normally only considered when there are many input variables and there is no linear relationship between the system’s inputs and outputs. In our case, data are acquired from several temperature sensors and the relation between the temperature values and phase values is not linear, according to Equation (6), which justifies the use of ML in the digital twin.

Neural networks are one way to build a digital twin model by using data, especially when a physics-based model is not accurate or even not available [5,6]. In this way, a neural network will be used as a first approach for the implementation of the digital twin. As data from the system accumulates, there is another approach that we will try: the use of Deep Neural Networks to detect patterns that are not visible to the user.

## 5. Implementing the Digital Twin

An accurate knowledge of a system’s behaviour is crucial to ensure the reliability and trustworthiness of the phase measurement system to be implemented. To help achieve these goals, we “replicated” the physical system into a digital counterpart. The schematics of the physical system up to the measure phase are represented in Figure 1.

The strategy to build the DT consists of modelling the transit time, obtaining a theoretical phase value that represents the passage of the modulated light in the fibre, and comparing this value with the phase measured by the multimeter.

Several factors that can influence the phase value will not be considered, e.g., the light modulation, the effect caused by the passage in the WDM, and the effects caused by fibre connections. The DT will focus solely on the propagation of light in the optical fibre and the phase variations caused by the temperature influence on the fibre path, on the lasers, on the photodetectors, and on the frequency mixer.

Before this work, several experiments were performed to calculate the effect of the temperature of the lasers and the detectors on the phase value, as well as the temperature influence of the mixer on the output voltage. These experiments are not included in this paper but will be presented in a later work. The values obtained for the temperature coefficients for the three pieces of equipment are presented in Table 1.

**Figure 2:**
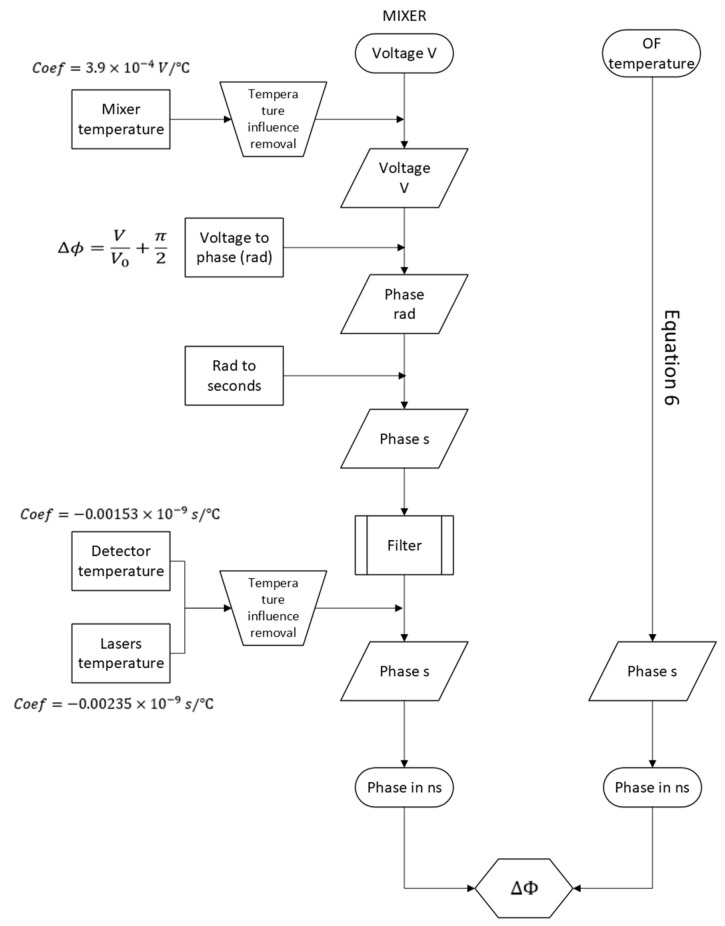
Schema for the calculation of the theoretical phase and experimental phase.

To transform the physical part of the system into its digital counterpart, we needed to convert the output of the mixer into time values, i.e., phase, and to compare this to the theoretical phase calculated from Equation (6). For this purpose, the schema from Figure 2 was used.

This modelling was divided in two parts: 1—computing a phase value from the mixer output; and 2—obtaining a phase value at the end of the optical fibre.

### 5.1. Phase from the Mixer Output

From the output voltage of the mixer, the effect of temperature on the mixer was added to the model; then, with Equation (1), the phase value was calculated in radians. This value was converted to seconds and filtered to eliminate higher-frequency noise. The temperature influence of the lasers and detectors was also added to the model, obtaining phase values in ns.

**Figure 3:**
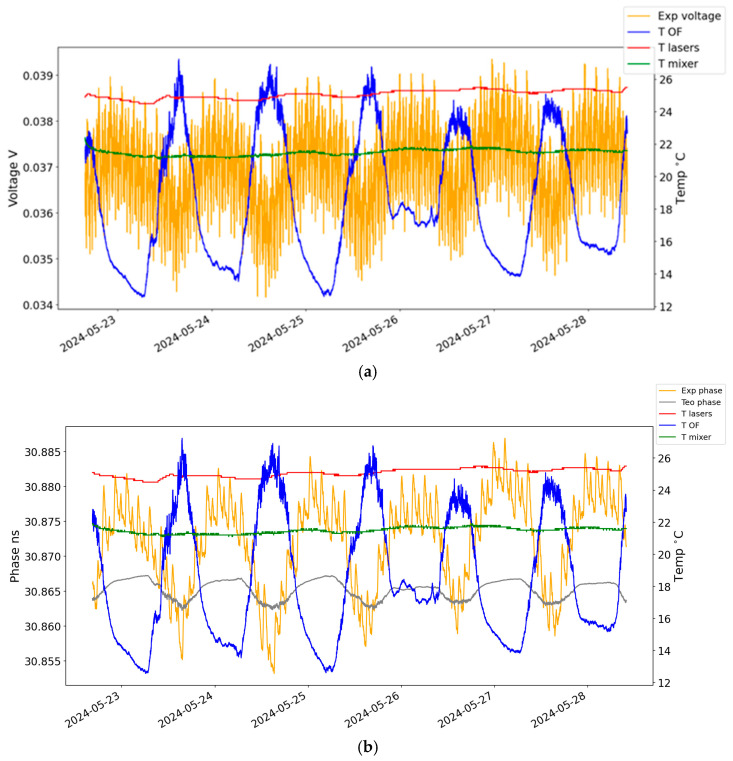
(**a**) Voltage values on the left axis and temperature values on the right axis. (**b**) Experimental and theoretical phase values on the left axis and temperature of the lasers, optical fibre, detectors, and mixer on the right axis.

This transformation can be observed in Figure 3, with the application of all steps mentioned above. In red, the temperature of the lasers is represented; in green, the temperature of the mixer and the detectors is represented; in blue, in chart (a), the temperature of the optical fibre is represented; in yellow, in chart (a), the voltage measured in the mixer is represented; and also in yellow, in chart (b), the processed phase obtained by the schema from Figure 2 is represented. A good agreement can be observed between the calculated values for phase and the temperature of the optical fibre.

### 5.2. Phase from the Optical Fibre

To calculate the theoretical differential phase delay, the procedure was the following: start with the measured temperature of the optical fibre (in blue, Figure 3a); apply it to Equation (6) to obtain the phase difference between the two signals traveling the optical fibre (in grey, Figure 3b).

The phase difference between the two values was calculated to be ∆Φ = 3.4 ns. For a better visualization, this gap was removed in Figure 3b. This discrepancy may be due to the different lengths of the “non-common” transmission paths. Specifically, these include the paths between the splitter, which divides the 10 MHz reference signal, and the WDM in the emission part, as well as the WDM and the phase meter inputs in the photodetector section. Additionally, the path difference between the photodetectors and the mixer, along with the copper connection cables from the detectors to the phase meter, may also contribute. These factors are not accounted for in the current digital twin model.

## 6. Estimation of the Measurement Uncertainty

One of the objectives of this work was to provide, as an output of the DT, a phase value with the associated uncertainty. All uncertainty components were identified and combined using the traditional approach of the Guide to the Expression of Uncertainty in Measurement, GUM [7], and the Monte Carlo method [8].

To estimate the uncertainty, several factors that contributed to the overall uncertainty were considered. The uncertainty associated with the measurement instruments was evaluated. For some equipment, calibration certificates were available, while for others, the uncertainty was estimated based on the literature and experiments conducted to assess the equipment’s performance, as was the case with the mixer. The mathematical model used for both approaches was based on Equation (6).

**Figure 4:**
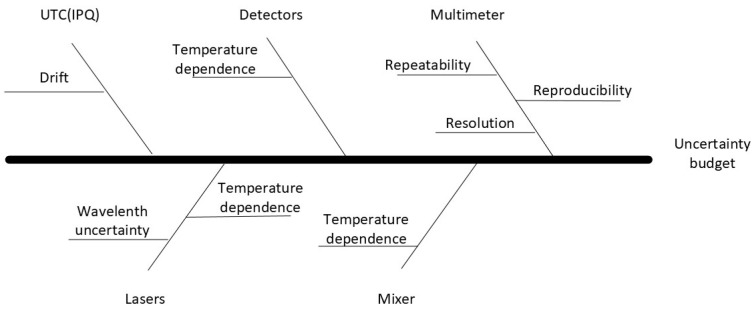
Determination of uncertainty budget using Ishikawa diagram.

For convenience, and to simplify the determination of the uncertainty budget considering the use of the mathematical model, the Ishikawa diagram was used, as shown in Figure 4.

**Figure 5:**
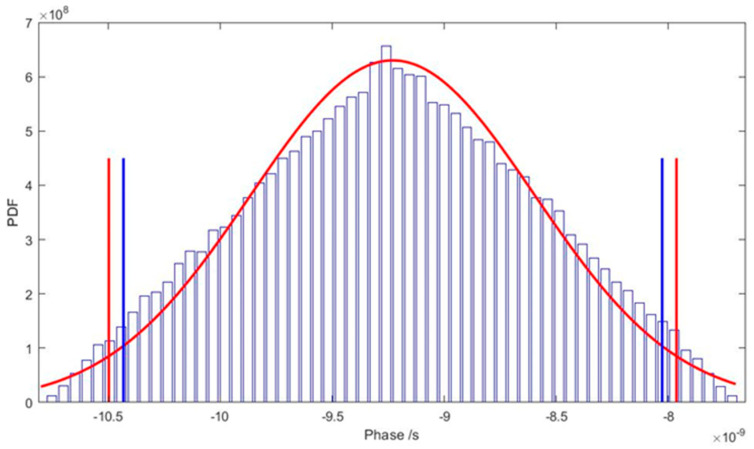
Chart of the comparison of uncertainty distribution. In blue is the Monte Carlo approach and in red is the GUM approach.

As mentioned above, the uncertainty was obtained by the GUM and by supplement 1 of the GUM. Both methods were used to validate the uncertainty, and the results obtained are represented in Figure 5 and Table 2. The Monte Carlo method is shown in blue and the GUM approach in red. The vertical lines represent the 95% confidence interval.

As recommended by the GUM, the uncertainty values shown in Table 2 have two significant digits, representing a difference of 10% between methods. Using more significant digits (1.201 × 10^−9^, 1.265 × 10^−9^) for the Monte Carlo and GUM, respectively, the difference falls to 5.3%, which represents a good agreement between the methods. Also, in the GUM approach, the Central Limit Theorem says that the combined uncertainty distribution converges to a normal distribution, which sometimes is not the case. In the Monte Carlo approach, the distribution of the combined uncertainty is determined by propagating the actual distributions of the input quantities and not merely their mean and standard deviation. In this case, since the distribution of the uncertainty is normal, according to Monte Carlo, and the obtained value, by the same method, is lower than that of the GUM approach, which overestimates the uncertainty in this case, we can conclude that the uncertainty calculation is validated.

## 7. DT Assembly and Test

The experimental data used as input for the digital twin were acquired over 6 days, using five temperature sensors and a multimeter. The multimeter used was a 34401A model from Agilent (Santa Clara, CA, USA) [9]. The temperature sensors were distributed along the external fibre path. A fourth temperature sensor was placed close to the laser source and a fifth one in the detector and mixer assembly. For the digital twin, temperature data were averaged from the three temperature sensors located outside. Figure 3a shows the acquired voltage values from the multimeter and the measured temperature values. At the instant of every voltage measurement, a temperature value from each sensor is also acquired.

To implement the digital twin, we used Python software Version 3.12, with several libraries and packages, e.g., Tensorflow Version 2.16.1, Keras Version 3, Numpy Version 1.26.1, and Pandas Version 3.1.3, just to mention a few [10].

The acquired data were transformed into a Pandas data frame, and the same preprocessing (data exploration) was performed on the raw data to search for missing data, outliers, and misclassifications.

After this process, the theoretical phase was calculated from the experimental values of the temperatures and added to the data frame as a new variable. The difference between this last variable and the experimental phase was also added as another variable. In this way, a data frame was built where some ML algorithms could be used. To train and validate the algorithm, the traditional hold-out sample method was used [5]. The data were divided into two parts to train the model and then validate and test it.

As mentioned previously, a neural network was used. In the process of building a neural network, one of the choices to make is what activation function should be used. The activation function decides whether a neuron should be activated or not by calculating the weighted sum and further adding bias to it. The purpose of the activation function is to introduce non-linearity into the output of a neuron, a process known as back-propagation [6,11]. Some tests were performed to evaluate the model’s accuracy with different activation functions, and the one chosen was a Sigmoid activation function $=\frac{1}{1+e^{-x}}$.

The training and validation process is an essential part of the process of creating machine learning models and is followed by the evaluation of the model’s accuracy. We used the Mean Absolute value of Error (MAE), which represents the difference between the original and predicted values, extracted by averaging the absolute difference over the data frame. The Mean Squared Error (MSE) is the square of the difference between the original and predicted values and represents the average difference over the data frame. These metrics were used to summarize and assess the quality of the machine learning model.

## 8. Results

With the experimental data represented in Figure 3, we trained two algorithms: a simple feedforward NN, also known as a Multilayer Perceptron with 64 neurons and a Sigmoid activation function, and a Long Short-Term Memory network (LSTM) with four memory cells and the Adam optimizer to adjust the model weights during training based on the computed gradients.

The LSTM network was chosen because these architectures are designed to work with sequences, making them ideal for tasks involving time or sequential data (e.g., time-series prediction or natural language processing) [12].

For the DT, the training focus was on the experimental phase, and the target was the difference between the theoretical phase and the experimental phase. After training, the training phase was added to the theoretical phase, being the output of the digital twin.

**Figure 6:**
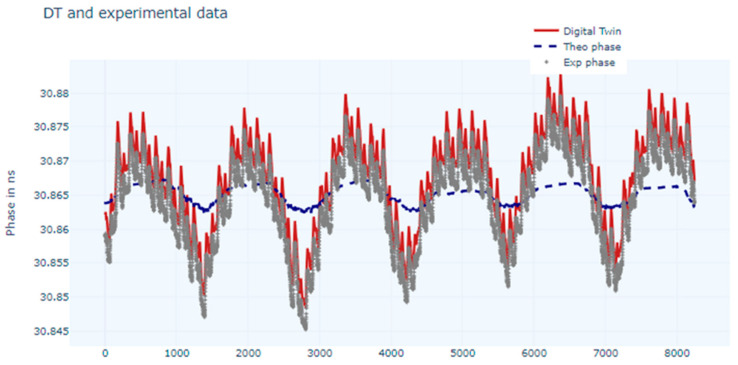
Chart of measured phase values, modelled values, and estimated values (digital twin) using a NN.

The digital twin training with the first NN can be observed in Figure 6, and we can see that the estimated phase follows the experimental data, which was the objective.

The metrics used to evaluate the performance of the algorithm were the Mean Absolute Error (MAE), which represents the average of the absolute difference between the actual and predicted values, i.e., measures the average of the residuals, and the Mean Squared Error (MSE), which represents the average of the squared difference between the original and predicted values, i.e., measures the variance of the residuals [6].

The values obtained were MAE = 0.006 and MSE = 5.1 × 10^−5^.

**Figure 7:**
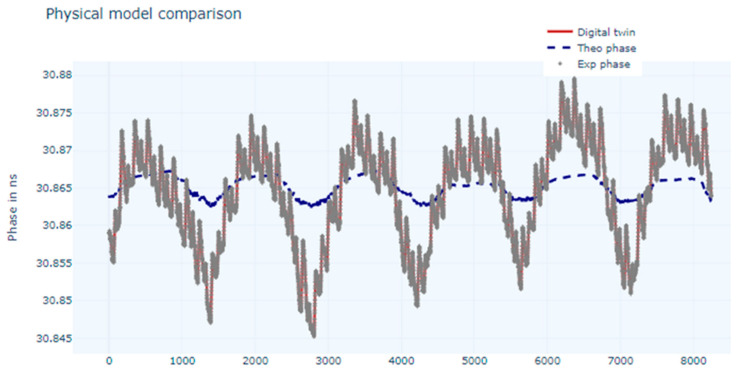
Chart for measured phase values, modelled values, and estimated values (digital twin) using LSTM network.

Training with the LSTM network gave the results presented in Figure 7.

The MAE and the MSE metrics obtained were 5.0 × 10^−3^ ns and 3.5 × 10^−5^ ns, respectively.

There is very good matching between the experimental data and the output of the DT for both the trained networks, but since the metrics are better in the case of the LSTM network, this one was chosen to train the DT model.

**Figure 8:**
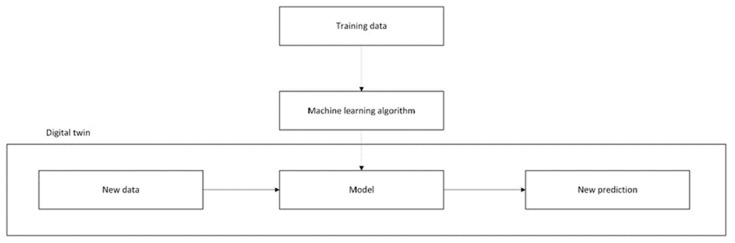
Schema for the digital twin.

For the implementation of the DT, the schema in Figure 8 was assumed, and to test the trained algorithm, a new set of data was acquired over 5 days.

**Figure 9:**
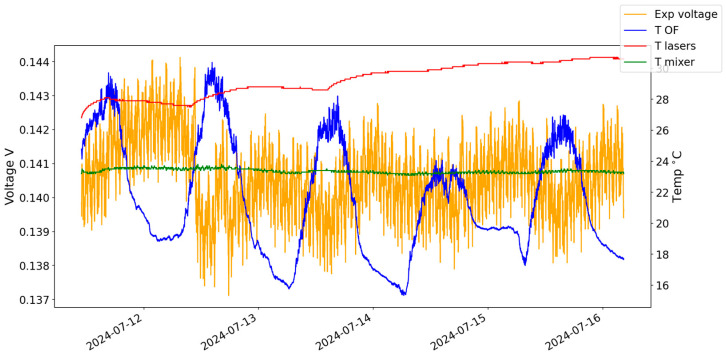
Five-day sequence of measurement data.

These 5 days of data acquisition are shown in Figure 9 and were treated in accordance with the schema in Figure 2.

**Figure 10:**
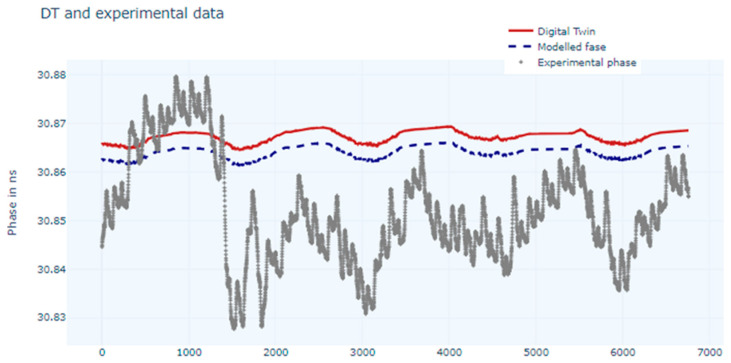
Trained model applied to theoretical data.

**Figure 11:**
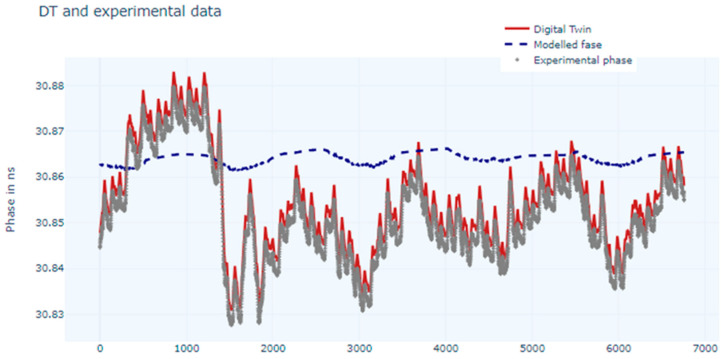
Trained model applied to experimental data.

The results of applying the model to this new set of data can be seen in Figure 10. The metrics obtained were 1.7 × 10^−2^ ns for the MAE and 3.6 × 10^−4^ ns for the MSE. The model was applied to the theoretical data and trained with the experimental data, which could explain the gap between the theoretical values and the predicted ones. This was not the case when we applied the model to the experimental data (Figure 11), where a good match was found, with a slight improvement in the metrics MAE and MSE of 1.7 × 10^−2^ ns and 3.5 × 10^−4^ ns. The implementation revealed that the digital twin will follow the experimental data more closely than the theoretical data. This could be an interesting argument in favour as experimental data integrate all influence factors, while theoretical data are reduced to a small set of variables.

## 9. Discussion/Conclusions

The digital twin, developed for use with a frequency mixer to detect phases, requires a combination of models and data to incorporate all the components of the physical model, as well as their respective uncertainty contributions.

This work presents a first attempt to train an algorithm focused on the use of a mixer to measure variations in the phases of two signals traveling over an optical fibre, allowing useful information to be obtained, leading to the correction factors that mitigate temperature effects. One of the benefits of the digital twin is that it will learn over time, with more data and with the introduction of other parameters of influence.

The implementation revealed that the digital twin aligns more closely with experimental data than theoretical data. This could be an interesting argument in favour as experimental data integrate all influence factors, while theoretical data are reduced to a small set of variables.

This digital twin includes all uncertainty contributions retrieved from the real data used for DT training and was estimated according to the GUM approach and validated with the Monte Carlo approach. The combined uncertainty of both methods with a difference of 5% shows a good agreement for both approaches.

However, the obtained uncertainty values are larger than the theoretical phase differences, as the combined uncertainty is in the order of ns, while the retrieved phase variations are in tens of ps. The main reason for this is that the main source of phase variation arises from the terminal equipment (lasers, detectors, mixer), and with lesser significance, from the light propagation in the fibre medium.

Nevertheless, for longer optical fibre paths, this difference will tend to be smaller as the terminal equipment influence is constant, while the propagation factor increases with fibre length.

The use of virtual experiments will allow the study of the overall effect of minor changes in some parts of the system that in “real life” could not be considered. As an example, we can see at some time points the effect of frequency transfer due to the long-term drift of some equipment.

The implementation of the digital twin, despite not covering a complete physical model (only the transmission process and phase detection with the mixer), nevertheless represents a good approach to digital twins that includes the totality of the physical model, as well as the lasers and photodetectors, as represented in Figure 1. The major influence during measurements is temperature variation, but other aspects need to be considered, such as laser stability, frequency modulation, photodetector accuracy, and other parameters of influence. The lasers used are diode lasers, and as temperature increases, the bandgap energy decreases, which shifts the emitted wavelengths to longer wavelengths; since the speed of light in the fibre is wavelength-dependent, changes in the temperature can influence the transit time of the light and the phase value at the input of the mixer. The accuracy of the photodetectors can change with temperature; dark current increases exponentially with temperature because thermal energy increases the rate of carrier generation. This creates a higher noise floor, reducing the signal-to-noise ratio and making it harder to detect low-amplitude signals, consequently reducing accuracy.

The evaluation of the model’s implementation and its application to new data was conducted by investigating the values obtained for MAE and MSE, which were close to zero, indicating the high accuracy of the model. The application of the model to new data revealed that the model should be applied to the experimental data, giving good concordance in values. We can assume that the LSTM network is a good choice for the algorithm.

We can conclude that using a frequency mixer as a phase detector, as represented in the DT, could be a viable solution for frequency transfer. It may be suitable for applications where the high level of accuracy typical of NMIs is not required. Although it can measure values in the range of tens of picoseconds, the resulting uncertainty is higher, in the order of nanoseconds. In future work, we want to compare, for small distances, this system to satellite-based systems used for frequency transfer, especially for small integration times.

As a concluding remark, we can add that this first implementation of a digital twin reveals its potential to be used as a low-budget system for phase detection using a mixer. A mixer less dependent on ambient temperature and more isolated to electric and magnetic effects would provide an output voltage with less noise, and in this way improve the accuracy of the measurements.

The data-based aspect, which is a major part of the DT, allows unknown parameters (not yet studied or discovered) to be compensated for in predictions.

We believe that the transformation of the physical part into the digital counterpart will allow the construction of a more robust digital twin that could eventually be adopted by NMIs (National Metrology Institutes) as a cost-effective solution for phase detection and frequency transfer.

## Figures and Tables

**Table 1:** Temperature coefficients measured for the lasers, detectors, and mixer.

	Lasers	Detectors	Mixer
Temperature Coefficient	$-0.00235\times 10^{-9}s/{}^{\circ}C$	$-0.00153\times 10^{-9}\;s/{}^{\circ}C$	$3.9\times 10^{-4\;}V/{}^{\circ}C$

**Table 2:** Values obtained for combined uncertainty.

Method	Combined Uncertainty
GUM	$1.3\times 10^{-9}$
Monte Carlo	$1.2\times 10^{-9}$

## Data Availability

The data presented in this study are available on request from the corresponding author.
